# Association of *IL4, IL6*, and *IL10* polymorphisms with pulmonary tuberculosis in a Tibetan Chinese population

**DOI:** 10.18632/oncotarget.23995

**Published:** 2018-01-06

**Authors:** Shumei He, Shenglai Yang, Qin Zhao, Liang Wang, Hang Liu, Yemeng Sheng, Dongya Yuan, Tianbo Jin

**Affiliations:** ^1^ Key Laboratory for Molecular Genetic Mechanisms and Intervention Research on High Altitude Disease of Tibet Autonomous Region, School of Medicine, Xizang Minzu University, Xianyang, Shaanxi 712082, China; ^2^ Key Laboratory for Basic Life Science Research of Tibet Autonomous Region, School of Medicine, Xizang Minzu University, Xianyang, Shaanxi 712082, China; ^3^ Key Laboratory of High Altitude Environment and Gene Related to Disease of Tibet Ministry of Education, School of Medicine, Xizang Minzu University, Xianyang 712082, Shaanxi, China; ^4^ Shaanxi Normal University, Xi’an, Shaanxi 710062, China; ^5^ Key Laboratory of Resource Biology and Biotechnology in Western China (Northwest University), Ministry of Education, Northwest University, Xi’an 710069, China

**Keywords:** pulmonary tuberculosis, IL4, IL6, IL10, Tibetan Chinese population

## Abstract

**Background:**

Pulmonary tuberculosis (PTB) is an infectious disease with a high incidence worldwide. Genes encoding cytokines *IL4*, *IL6*, and *IL10* are highly polymorphic and can influence the susceptibility to PTB.

**Results:**

We found correlations between one SNP in IL6 (rs2069837 *p* = 6.63E-11), seven SNPs in *IL10* (rs1554286 *p* = 6.87E-20, rs1518111 *p* = 6.11E-11, rs3021094 *p* = 6.75E-29, rs3790622 *p* = 2.40E-06, rs3024490 *p* = 6.73E-11, rs1800872 *p* = 6.18E-11, rs1800871 *p* = 6.73E-11) and incidences of PTB. The SNPs rs2069837, rs1554286, rs1518111, rs3024490, rs1800872, and rs1800871 increased PTB risk by 1.95-fold, 2.34-fold, 1.84-fold, 1.84-fold, 1.84-fold and 1.84-fold, respectively. The SNPs rs3021094 and rs3790622 decreased PTB risk by 0.33-fold and 0.38-fold, respectively. We also found two linkage disequilibrium blocks in the studied *IL* SNPs. The *IL4* haplotype TCCCGGA (OR = 1.33, *p* = 0.014) increased PTB risk, the *IL10* haplotypes ATGGATA (OR = 0.39, *p* = 4.84E-06) provided a protective effect and decreased PTB risk.

**Materials and Methods:**

For this study, we recruited 467 subjects with PTB and 503 healthy subjects from a Tibetan population living in Lhasa and nearby, China. Association analyses of sixteen single-nucleotide polymorphisms (SNPs) in IL4, IL6, and *IL10* were performed.

**Conclusions:**

Our findings demonstrate an association between polymorphisms in *IL6* and *IL10* and risk of PTB.

## INTRODUCTION

Pulmonary tuberculosis (PTB) is caused by *Mycobacterium tuberculosis* (*M. tuberculosis*) and is one of the leading causes of death worldwide. Since 1980, the incidences of PTB and mortality rates have increased rapidly [1]. Tuberculosis in 2009 global statistics show that about 9.4 million people are infected with tuberculosis, 1.7 million people died of tuberculosis [2]. The incidence of PTB in Asian countries accounts for 60% of the total infections worldwide. The number of PTB patients in China is the second largest in the world, and China is considered as one of the 22 high-burden countries. Tibet is one of the regions with high TB burden. The statistical analysis of the tuberculosis epidemic situation in the Tibet Autonomous Region in 2011–2013 showed that the number of cases and incidence of tuberculosis in Tibet Autonomous Region was 4046 and 134.9/100,000, 4096 and 136.5/100,000, 4273 and 142.4/100,000, respectively. Although *M. tuberculosis* has infected almost one-third of the population worldwide, only 10% of patients produce corresponding clinical symptoms during their lifetime [3], implicating that additional factors may influence the incidence of disease among different individuals.

Previous studies have identified genes that confer disease susceptibility by regulating the immune response [4, 5]. A twin study found that PTB concordance in identical twins is 2-fold higher than in non-identical twins [6]. Thus, we expect that identification of host genetic factors for PTB susceptibility may play a key role in PTB control worldwide. The first published tuberculosis GWAS identified chr18q11 rs4331426 as the susceptible loci for tuberculosis [7]. After that, in Garner, Gambia, Indonesia, and Russia, rs2057178, located on the chr11q3 downstream of the WT1 gene, was identified as a resistance gene loci [8]. In a recent GWAS study published in the Russian population, the ASAP1 gene intron SNP (rs4733781, rs10956514) located on the 8q24 chromosome was associated with tuberculosis [9]. In the Chinese population validation study found that the results differ from those of other ethnic groups. In recent years, more and more research has been done on the susceptibility to tuberculosis in the world. Linkage analysis and candidate gene association studies identified a number of possible susceptibility genes for tuberculosis, which can be divided into human leukocyte associated antigen genes and non HLA genes. Non HLA genes include *VDR*, *MBL*, *TLR*, *P2X*, *IL4*, *IL6, IL 10,* and so on [10–14].

Interleukin-4 (*IL-4*), is located on chromosome 5q31.1, an anti-inflammatory cytokine has been implicated to down-regulate IFN-γ, and thus has a deleterious effect on TB patients [13, 15]. One research found that *IL4 variant* rs2070874 showed a two-fold risk by T allele in north Indians (OR = 1.8, *p* = 0.01) [13]. In Iranians, *IL-4* -590 (rs2243250) C allele and the TC genotypes were found to be significantly more common in TB patients than in controls, and the -1098 (rs2243248) and -33 (rs2070874) were not found to be associated with susceptibility to TB [16]. Qi H et al. [15] investigated the association between susceptibility to TB and single-nucleotide polymorphisms (SNPs) of the *IL-4* (rs2243250, rs2243268, rs2243274, and rs2243282) and IL-10 genes (rs1800871, rs3021094, and rs3790622) in Chinese population, and found that rs2243268-A and rs2243274-G of the *IL-4* gene reduced the risk of developing EPTB and severe TB.

*IL-6* is secreted by Toll-like receptor 2-expressing cells in response to the presence of Mycobacterium tuberculosis early in infection and is involved in anti-tuberculosis immunity in the body [17]. High levels of IL-6 cytokine are produced in response to MTB infection [18], and its role seems especially critical when bacterial burden is high [19]. Rong H et al. [20] shown that rs1524107 CT genotype significantly increased the risk of TB, but the association disappeared after stratified by age and gender.

Interleukin 10 (*IL10*) is a multifunctional cytokine mainly produced by Th2 cells, the main function of antigen presentation function for stromal macrophages, through down-regulation of inflammatory cytokines, thereby inhibiting macrophage function during inflammation, inhibit Th1 cell response, inhibition of Th1 cells to produce cytokines IFN-γ, is a kind of cytokines the incidence of tuberculosis, play a role in the pathogenesis of tuberculosis. The study found that IL10 gene associated with the risk of tuberculosis [21]. Studies in Asian population reported the minor allele “G” of rs1800172 in *IL10* was associated with a decreased PTB risk (OR = 0.69) whereas no risk association was found in Europeans (OR = 1.19), Africans (OR = 1.01) [22]. The study of Halstrom S implicated the minor (A) allele of rs1518111 in risk of pulmonary NTM disease in Caucasians [23].

Through the literature search, we found that interleukin gene is related to the occurrence of tuberculosis, few studies have explored the association between *IL4*, *IL6,* and *IL10* polymorphisms and PTB risk in Chinese populations. So we selected *IL4*, *IL6*, *IL10* genes as the study. We combined our previous literature, selected seven *IL4* SNPs, two *IL6* SNPs, and seven *IL10* SNPs to evaluate whether single-nucleotide polymorphisms (SNPs) in the three genes were associated with PTB risk in a Tibetan Chinese population.

## RESULTS

We recruited 467 cases, average age 50.67 ± 7.80 years, including 287 female and 180 male; also recruited 503 cases, average age 50.34 ± 7.74 years, including 308 female and 195 male. As listed in Table 1, the *p* value of age (*p* = 0.686) and sex (*p* = 0.947) was more than 0.05, respectively.

**Table 1:** Patient demographics

Gender	case (467)	control (503)	*p*
female	287	308	0.947^a^
male	180	195	
Age, year	50.67 ± 7.80	50.34 ± 7.74	0.686^b^

^a^*p* values was calculated from Pearson’s chi-square tests.

^b^*p* values was calculated by Welch’s *t* tests.

*p* < 0.05 indicates statistical significance.

An overview (MAF, OR, 95% CI, position, HWE, band, alleles, *p*-value) for all candidate SNPs for the study participants was made (Table 2). One SNP rs1524107 (*p* = 2.79E-140) was deviated from Hardy–Weinberg Equilibrium (*p <* 0.05) and was excluded in our analysis. Upon investigating the sixteen SNPs in *IL4*, *IL6*, and *IL10* (Table 2), we found one SNP in *IL6* (rs2069837: OR = 1.95, 95% CI = 1.60-2.39, *p* = 6.63E-11) and five SNPs in *IL10* (rs1554286: OR = 2.34, 95% CI = 1.95–2.81, *p* = 6.87E-20; rs1518111: OR = 1.84, 95% CI = 1.53–2.22, *p* = 6.11E-11; rs3024490: OR = 1.84, 95% CI = 1.53–2.21, *p* = 6.73E-11; rs1800872: OR = 1.84, 95% CI = 1.53–2.22, *p* = 6.18E-11; and rs1800871: OR = 1.84, 95% CI = 1.53–2.21, *p* = 6.73E-11) that were correlated with increased PTB risk after the Bonferroni correction. We also found two SNPs in *IL10* (rs3021094: OR = 0.33, 95% CI = 0.27–0.40, *p* = 6.75E-29; and rs3790622: OR = 0.38, 95% CI = 0.25–0.58, *p* = 2.40E-06) that were correlated with decreased PTB risk after the Bonferroni correction.

**Table 2:** Summary of basic *IL* SNP information for all study participants

SNP_ID	Gene	Band	MAF		Alleles Aa/B	Role	HWE-p	OR (95% CI)	*p*-value
			Case	Control					
rs2070874	*IL4*	5q31.1	0.184	0.223	C/T	5′UTR	0.093	0.79 (0.63–0.99)	0.035
rs2227282	*IL4*	5q31.1	0.150	0.166	G/C	Intron	0.423	0.89 (0.69–1.13)	0.331
rs2243267	*IL4*	5q31.1	0.184	0.224	G/C	Intron	0.073	0.78 (0.62–0.98)	0.031
rs2243268	*IL4*	5q31.1	0.184	0.224	A/C	Intron	0.073	0.78 (0.62–0.98)	0.031
rs2243270	*IL4*	5q31.1	0.184	0.224	A/G	Intron	0.073	0.78 (0.62–0.98)	0.031
rs2243288	*IL4*	5q31.1	0.185	0.221	A/G	Intron	0.119	0.80 (0.64–1.01)	0.053
rs2243290	*IL4*	5q31.1	0.183	0.217	C/A	Intron (boundary)	0.089	0.81 (0.64–1.01)	0.065
rs2069837	*IL6*	7p15.3	0.345	0.213	G/A	Intron	1	1.95 (1.60–2.39)	6.63E-11^*^
rs1524107	*IL6*	7p15.3	0.225	0.427	T/C	Intron (boundary)	2.79E-140^#^	0.39 (0.32–0.47)	3.30E-21^*^
rs1554286	*IL10*	1q32.1	0.535	0.330	G/A	Intron (boundary)	0.420	2.34 (1.95–2.81)	6.87E-20^*^
rs1518111	*IL10*	1q32.1	0.473	0.328	C/T	Intron (boundary)	0.156	1.84 (1.53–2.22)	6.11E-11^*^
rs3021094	*IL10*	1q32.1	0.217	0.457	G/T	Intron	0.178	0.33 (0.27–0.40)	6.75E-29^*^
rs3790622	*IL10*	1q32.1	0.034	0.085	A/G	Intron	1	0.38 (0.25–0.58)	2.40E-06^*^
rs3024490	*IL10*	1q32.1	0.469	0.324	C/A	Intron	0.309	1.84 (1.53–2.21)	6.73E-11^*^
rs1800872	*IL10*	1q32.1	0.469	0.324	G/T	Promoter	0.264	1.84 (1.53–2.22)	6.18E-11^*^
rs1800871	*IL10*	1q32.1	0.469	0.324	G/A	Promoter	0.309	1.84 (1.53–2.21)	6.73E-11^*^

SNP: single-nucleotide polymorphism; MAF: minor allele frequency; HWE: Hardy-Weinberg equilibrium; OR: odds ratio; CI: confidence interval; ^a^: Minor allele.

^*^p < 0.05 indicates statistical significance.

^#^SNP was excluded in the subsequent analysis for deviated from Hardy–Weinberg Equilibrium (*p <* 0.05).

The effects of interleukin gene (*IL4*, *IL6*, *IL10*) on tuberculosis were assessed by logistic regression analysis under three genetic models, including dominant, recessive and log-additive models (Table 3). For rs2069837 in *IL6*, found that the risk of tuberculosis in individuals carrying risk allele G was 1.88 times in log-additive model (OR = 1.88, 95% CI = 1.54–2.30, *p* = 6.83E-10). For rs1554286 in *IL10*, we found that the minor allele “G” raised the risk of PTB under the log-additive model (OR = 2.28, 95% CI = 1.89–2.75, *p* = 1.07E-17). For rs1518111 in *IL10*, under the log-additive model, we found that individuals with risk allele C had a greater risk of tuberculosis than carrying wild alleles increased by 79% (OR = 1.79, 95% CI = 1.49–2.14, *p* = 5.04E-10). For rs3021094 in *IL10*, we found that the minor allele “G” protect the risk of tuberculosis in the log-additive model (OR = 0.34, 95% CI = 0.28–0.42, *p* = 3.31E-24).

**Table 3:** Association between *IL* gene polymorphism and PTB risk

SNP_ID	Model	Genotype	Control	Case	OR (95% CI)	*p*-value
rs2069837	Dominant	A/A	312 (62.03%)	211 (45.28%)	1	1.98E-07^*^
		G/G-G/A	191 (37.97%)	255 (54.72%)	1.97 (1.53–2.55)	
	Recessive	G/A-A/A	480 (95.43%)	399 (85.62%)	1	5.84E-07^*^
		G/G	23 (4.57%)	67 (14.38%)	3.50 (2.14–5.73)	
	Log-additive	---	---	---	1.88 (1.54–2.30)	6.83E-10^*^
rs1554286	Dominant	A/A	230 (45.73%)	104 (22.27%)	1	4.18E-14^*^
		G/G-G/A	273 (54.27%)	363 (77.73%)	2.94 (2.22–3.89)	
	Recessive	G/A-A/A	444 (88.27%)	330 (70.66%)	1	3.38E-11^*^
		G/G	59 (11.73%)	137 (29.34%)	3.12 (2.23–4.38)	
	Log-additive	---	---	---	2.28 (1.89–2.75)	1.07E-17^*^
rs1518111	Dominant	T/T	234 (46.61%)	135 (28.91%)	1	1.80E-08^*^
		C/C-C/T	268 (53.39%)	332 (71.09%)	2.15 (1.65–2.82)	
	Recessive	C/T-T/T	441 (87.85%)	357 (76.45%)	1	4.60E-06^*^
		C/C	61 (12.15%)	110 (23.55%)	2.23 (1.58–3.14)	
	Log-additive	---	---	---	1.79 (1.49–2.14)	5.04E-10^*^
rs3021094	Dominant	T/T	156 (31.01%)	287 (61.59%)	1	6.38E-21^*^
		G/G-G/T	347 (68.99%)	179 (38.41%)	0.28 (0.22–0.37)	
	Recessive	G/T-T/T	390 (77.53%)	443 (95.06%)	1	6.38E-13^*^
		G/G	113 (22.47%)	23 (4.94%)	0.18 (0.11–0.29)	
	Log-additive	---	---	---	0.34 (0.28–0.42)	3.31E-24^*^
rs3790622	Dominant	G/G	420 (83.50%)	435 (93.15%)	1	6.44E-06^*^
		A/A-A/G	83 (16.50%)	32 (6.86%)	0.37 (0.24–0.57)	
	Recessive	A/G-G/G	500 (99.40%)	467 (100%)	1	0.999
		A/A	3 (0.6%)	0 (0%)	-	
	Log-additive	---	---	---	0.37 (0.24–0.57)	4.60E-06^*^
rs3024490	Dominant	A/A	235 (46.72%)	134 (28.69%)	1	9.81E-09^*^
		C/C-C/A	268 (53.28%)	333 (71.31%)	2.18 (1.67–2.84)	
	Recessive	C/A-A/A	445 (88.47%)	362 (77.52%)	1	7.20E-06^*^
		C/C	58 (11.53%)	105 (22.48%)	2.23 (1.57–3.16)	
	Log-additive	---	---	---	1.81 (1.50–2.17)	3.75E-10^*^
rs1800872	Dominant	T/T	235 (46.81%)	134 (28.69%)	1	8.45E-09^*^
		G/G-G/T	267 (53.19%)	333 (71.31%)	2.19 (1.68–2.88)	
	Recessive	G/T-T/T	444 (88.45%)	362 (77.52%)	1	7.65E-06^*^
		G/G	58 (11.55%)	105 (22.48%)	2.22 (1.57–3.15)	
	Log-additive	---	---	---	1.81 (1.50–2.17)	3.54E-10^*^
rs1800871	Dominant	A/A	235 (46.72%)	134 (28.69%)	1	9.81E-09^*^
		G/G-G/A	268 (53.28%)	333 (71.31%)	2.18 (1.67–2.84)	
	Recessive	G/A-A/A	445 (88.47%)	362 (77.52%)	1	7.20E-06^*^
		G/G	58 (11.53%)	105 (22.48%)	2.23 (1.57-3.16)	
	Log-additive	---	---	---	1.81 (1.50–2.17)	3.75E-10^*^

SNP: single-nucleotide polymorphism; ORs: odds ratio; CI: confidence interval.

^*^p < 0.05 indicates statistical significance.

For the rs3790622 loci, the specific results showed that the risk of pulmonary tuberculosis in individuals with AA-AG genotype was 63% lower than that of wild type individuals under dominant genetic model (OR = 0.37, 95% CI = 0.24–0.57, *p* = 6.44E-06). Similarly, we also found that the risks of tuberculosis in individuals with *IL10* rs3024490 C allele, rs1800872 G allele and rs1800871 G allele were 1.81 times, 1.81 times and 1.81 times than the individuals with wild type (rs3024490: OR = 1.81, 95% CI = 1.50–2.17, *p* = 3.75E-10; rs1800872: OR = 1.81, 95% CI = 1.50–2.17, *p* = 3.54E-10; rs1800871: OR = 1.81, 95% CI = 1.50–2.17, *p* = 3.75E-10).

**Figure 1:**
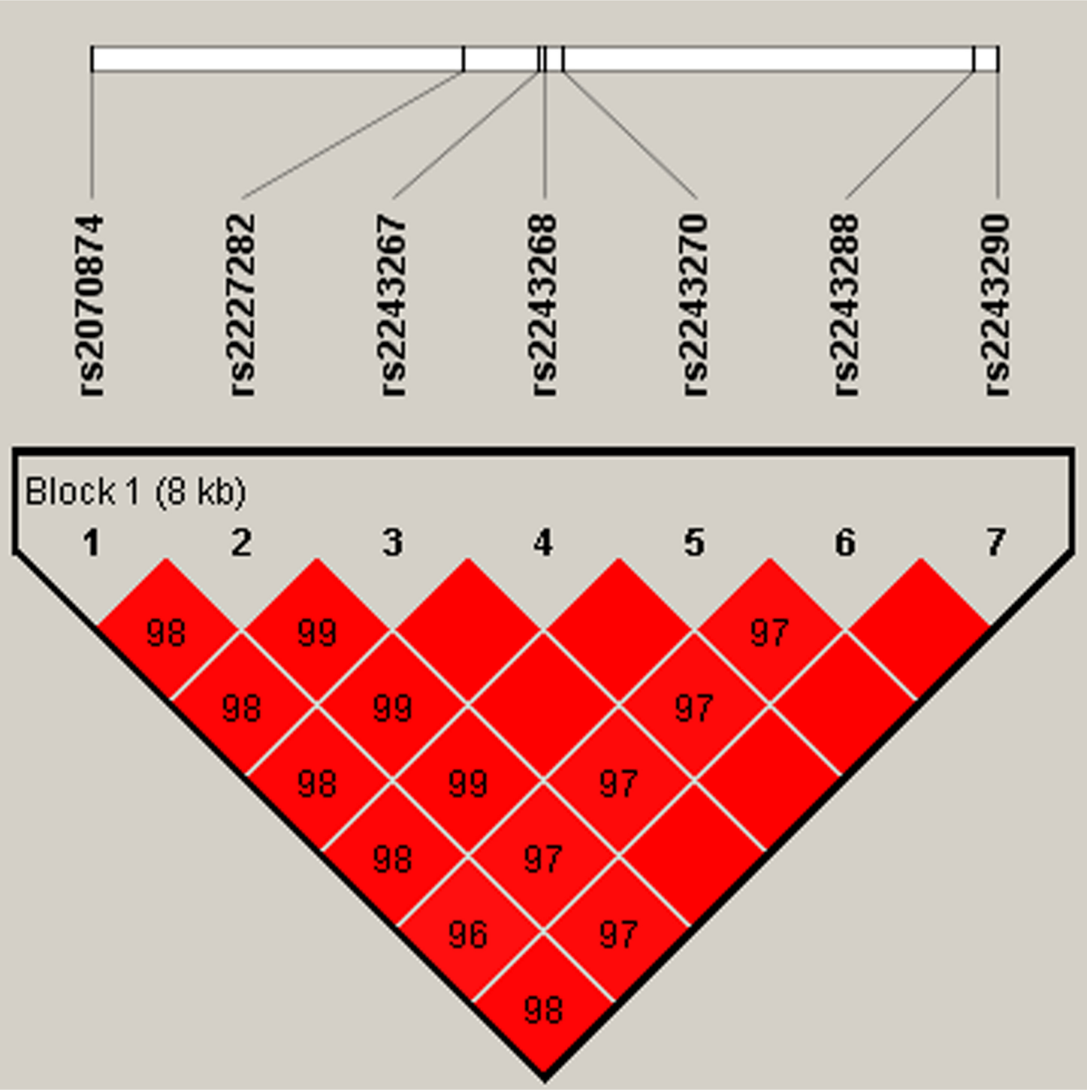
Haplotype block for the SNPs of IL4 A standard color scheme is used to display LD with bright red for very strong LD (LOD = 2, D’ = 1).

**Figure 2:**
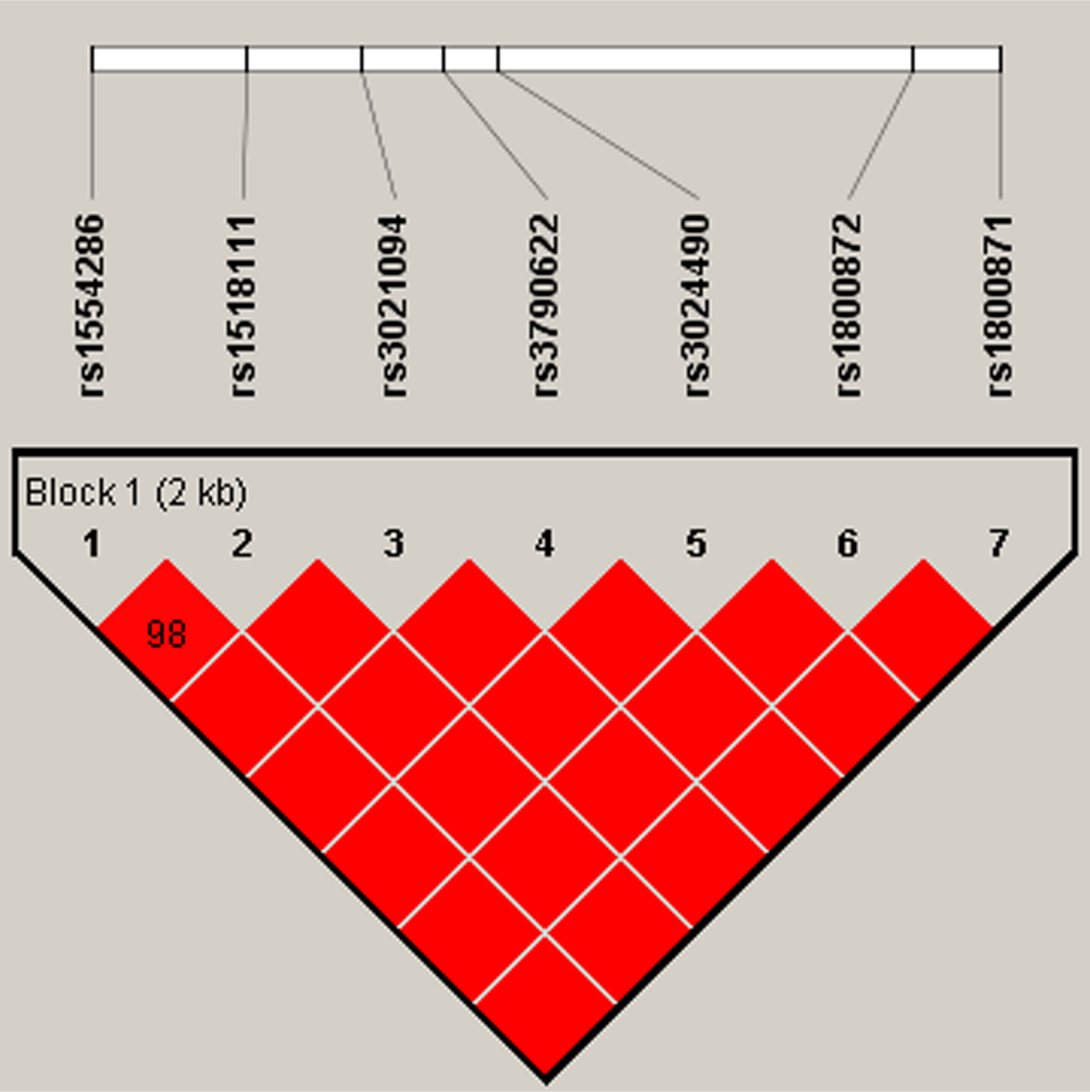
Haplotype block for the SNPs of IL10 A standard color scheme is used to display LD with bright red for very strong LD (LOD = 2, D’ = 1).

In this research, we used SHEsis software platform to do haplotype analysis. In our result, a block in *IL4* was constructed by rs2070874, rs2227282, rs2243267, rs2243268, rs2243270, rs2243288, and rs2243290 in chromosome 5 with D’>0.8, among them the D’ of rs2243267 and rs2243268, rs2243267 and rs2243270, rs2243268 and rs2243270, rs2243267 and rs2243290, rs2243268 and rs2243290, rs2243270 and rs2243290, and rs2243288 and rs2243290 was 1, respectively (Figure 1). A block in *IL10* constructed by rs1554286, rs1518111, rs3021094, rs3790622, rs3024490, rs1800872, and rs1800871 in chromosome 1 with D’>0.8, among them the D’ of rs1554286 and rs1518111 was 0.98, the D’ of the other loci reach 1 (Figure 2). Association analyses between the three blocks and risk of PTB were performed (Table 4). In *IL4*, the haplotype “T_rs2070874_C_rs2227282_C_rs2243267_C_rs2243268_G_rs2243270_G_rs2243288_A_rs2243290_” (OR = 1.33, 95% CI = 1.06–1.67, *p* = 0.014) was associated with increased PTB risk. The haplotype “C_rs2070874_C_rs2227282_G_rs2243267_A_rs2243268_A_rs2243270_A_rs2243288_C_rs2243290_” was associated with decreased PTB risk (OR = 0.64, 95% CI = 0.41–1.00, *p* < 0.049). In *IL10*, the haplotypes “G_rs1554286_T_rs151811_T_rs3021094_G_rs3790622_A_rs3024490_T_rs1800872_A_rs1800871_” (OR = 11.51, 95% CI = 4.94–26.86, *p* = 1.56E-08) and “G_rs1554286_C_rs151811_T_rs3021094_G_rs3790622_C_rs3024490_G_rs1800872_ G_rs1800871_” (OR = 1.80, 95% CI = 1.50–2.17, *p* = 3.97E-10) were associated with increased PTB risk. The haplotypes “A_rs1554286_T_rs151811_G_rs3021094_G_rs3790622_A_rs3024490_T_rs1800872_ A_rs1800871_” (OR = 0.39, 95% CI = 0.31-0.48, *p* = 6.97E–18) and “A_rs1554286_T_rs151811_G_rs3021094_ A_rs3790622_A_rs3024490_T_rs1800872_A_rs1800871_” (OR = 0.37, 95% CI = 0.24–0.57, *p* = 4.84E-06) were associated with decreased PTB risk.

**Table 4:** Haplotype analysis results for *IL* SNPs

Block ID	Haplotype	Frequency (%)		OR (95% CI)	*p*-value
		Case	Control		
*IL4*	rs2070874|rs2227282|rs2243267|rs2243268|rs2243270|rs2243288|rs2243290				
	C G G A A A C	14.99	16.30	0.90 (0.70–1.16)	0.414
	T C C C G G A	81.37	76.94	1.33 (1.06–1.67)	0.014^*^
	C C G A A A C	3.32	5.17	0.64 (0.41–1.00)	0.049^*^
*IL10*	rs1554286|rs1518111|rs3021094|rs3790622|rs3024490|rs1800872|rs1800871				
	A T G G A T A	18.24	37.18	0.39 (0.31–0.48)	6.79E-18^*^
	G T T G A T A	6.65	0.60	11.51 (4.94–26.86)	1.56E-08^*^
	G C T G C G G	46.89	32.41	1.80 (1.50–2.17)	3.97E-10^*^
	A T T G A T A	24.25	20.97	1.20 (0.97–1.49)	0.087
	A T G A A T A	3.43	8.55	0.37 (0.24–0.57)	4.84E-06^*^

SNP: single-nucleotide polymorphism; OR: odds ratio; CI: confidence interval.

^*^p < 0.05 indicates statistical significance.

## DISCUSSION

In this study, we investigated the association between *IL* polymorphisms and PTB risk in a Tibetan Chinese population. We found one SNP in *IL6* and five SNPs in *IL10* that were associated with increased PTB risk: rs2069837 (A>G; OR = 1.95), rs1554286 (A>G; OR = 2.34), rs1518111 (T>C; OR = 1.84), rs3024490 (A>C; OR = 1.84), rs1800872 (T>G; OR = 1.84) and rs1800871 (A>G; OR = 1.84), and two SNPs in *IL10* that were associated with decreased PTB risk: rs3021094 (T>G; OR = 0.33) and rs3790622 (G>A; OR = 0.38).

Previous PTB studies investigated the function of host genetic factors and immune response in *M. tuberculosis* (MTB) infection [24]. In humans, macrophages are the main cells involved in intracellular replication of MTB. Macrophages also serve as antigen-presenting cells during the reactivation of lymphocytes, and they function as a vital killer of mycobacteria [25]. Cytokines such as *IL6* and *IL10* play a key role in driving the appropriate immune response against mycobacteria via activation of inflammatory and immunomodulatory networks orchestrated by both macrophages and T cells [26]. The importance of cytokine *IL6* has been studied in previous studies demonstrating that *IL6* is important for optimal T-cell development [27, 28]. High levels of *IL6* are produced in response to MTB infection and *IL6* is critical when bacterial burden is high [19, 29, 30]. A study done in a Southern Brazilian population found that the minor allele “C” of rs1800795 in *IL6* decreased the risk of PTB development (OR = 0.49, 95% CI: 0.31–0.78, *p* = 0.008) [12].

Interestingly, more recent meta-analysis studies of rs1800896 in *IL10* revealed conflicting results in an analysis of the European and American population [21, 22]. Studies in Asian population reported the minor allele “G” of rs1800172 in *IL10* was associated with a decreased PTB risk (OR = 0.69, 95% CI: 0.31-0.78, *p* < 0.01) whereas no risk association was found in Europeans (OR = 1.19, 95% CI: 0.92-1.54, *p* = 0.18), Africans (OR = 1.01, 95% CI: 0.92-1.10, *p* = 0.90) [22]. Haplotype analysis found polymorphisms “GCC” constructed by -1082G/A, -592 A>C and -819 C>T in the *IL10* promoter region was in perfect linkage disequilibrium and was associated with an increased PTB risk (GCC vs. others: *p* = 1.42, 95% CI = 1.02-1.97, *p* = 0.04) [21].

*IL6* and *IL10* are both cytokines that function in the inflammation process. To our knowledge, our study is the first to investigate the association between polymorphic SNPs of three *IL* genes and PTB risk in a Tibetan Chinese population. Our findings demonstrate an association between polymorphisms in IL6 and IL10 and risk of PTB. We speculate that *IL6* and *IL10* polymorphisms might modify gene expression and biological function. Polymorphisms of *IL6* and *IL10* that influence the ability of macrophages to kill *M. tuberculosis* in PTB patients may be a risk factor for PTB. And in the further, we will conduct further validation and disease prediction studies.

## MATERIALS AND METHODS

### Ethics statement

Our present study strictly followed the principles of the Declaration of Helsinki of the World Medical Association and was approved by the Ethics Committee of School of Medicine, Xizang Minzu University. Informed consent forms were signed by all participants.

### Study participants

A total of 467 Tibetan PTB patients were recruited from the third Hospital of Tibet Autonomous Region from October 2012 to September 2013. Patients were diagnosed based on the criteria of sputum smear or culture positive for MTB pathogen, clinical-radiological findings, and histological evidence of PTB. We excluded individuals who were infected with nontuberculous Mycobacterium, treated with immunosuppressants, or exhibited only extrapulmonary tuberculosis without PTB. At the same time, stochastic samples of 503 healthy controls were enlisted from the same geographical origin and were living in the same region as the patients with PTB. The controls had no previous clinical history or laboratory criteria suggestive of PTB infection. All subjects were all Tibetan Chinese living in Lhasa and nearby.

Cases and controls had at least three generations of paternal ancestry in their ethnic. Cases and controls with any of the following conditions were excluded from the study: human immunodeficiency virus (HIV) positive or known to have any autoimmune, chronic inflammatory, or other diseases.

### SNP selection

Through the literature search, we found that interleukin gene is related to the occurrence of tuberculosis, so we selected IL4, IL6, IL10 genes as the study. We combined our previous literature [13, 15, 20, 23, 31, 32] [15, 20, 23, 32–34], selected seven *IL4* SNPs, two *IL6* SNPs, and seven *IL10* SNPs with a minor allele frequency (MAF) above 5% and r^2^ greater than 0.8 from the HapMap Chinese Han Beijing population.

### Genotyping

Genomic DNA was extracted from peripheral blood samples using a genomic DNA purification kit (GoldMag, Xi’an, China). We used spectrometry (DU530 UV/VIS spectrophotometer, Beckman Instruments, Fullerton, CA) to measure the DNA concentration. The primers for amplification and extension reactions were designed with Sequenom MassARRAY Assay Design 3.0 Software (Sequenom, San Diego, CA) [33]. We used Sequenom MassARRAY RS1000 to perform the SNP genotyping with the agreement of the manufacturer [33, 35], and we used Sequenom Typer 4.0 software for data management and analysis [33, 34].

### Statistical analysis

Microsoft Excel (Microsoft, Redmond, WA) and SPSS Statistics (version 17.0, SPSS, Chicago, IL) were used for statistical analyses. All *p*-values were two-tailed, and *p* < 0.05 was considered to be statistically significant. SNP genotype frequencies in the case and control groups were calculated by Chi-square tests and the Hardy-Weinberg equilibrium (HWE) was used to check the genotype frequency of the control group. Unconditional logistic regression analysis was used to examine the odds ratios (ORs) and 95% confidence intervals (CIs) in order to assess the association between SNPs and PTB [35]. Three models (dominant, recessive, log-additive) were used to test the association between SNPs and PTB [36]. Furthermore, Haploview (version 4.2, Broad Institute, Cambridge, MA) and SHEsis software (http://www.nhgg.org/analysis/) were used to check the linkage disequilibrium (LD), haplotype construction, and genetic association at polymorphism loci. A D’ value greater than 0.8 indicated that the related SNPs formed one block [37].
